# Integrative Analysis of Global Gene Expression Identifies Opposite Patterns of Reactive Astrogliosis in Aged Human Prefrontal Cortex

**DOI:** 10.3390/brainsci8120227

**Published:** 2018-12-19

**Authors:** César Payán-Gómez, Diego Rodríguez, Diana Amador-Muñoz, Sandra Ramírez-Clavijo

**Affiliations:** 1 Facultad de Ciencias Naturales y Matemáticas, Universidad del Rosario, Bogotá 111221, Colombia; sandra.ramirez@urosario.edu.co; 2 Neuroscience (NEUROS) Research Group, School of Medicine and Health Sciences, Universidad del Rosario, Carrera 24 No. 63C-69, Bogotá 111221, Colombia; diegoalejan.rodrig01@urosario.edu.co (D.R.); diana.amador@urosario.edu.co (D.A.-M.)

**Keywords:** Prefrontal cortex aging, meta-analysis of transcriptomic, synapsis aging, reactive astrogliosis

## Abstract

The prefrontal cortex (PFC) is one of the brain regions with more prominent changes in human aging. The molecular processes related to the cognitive decline and mood changes during aging are not completely understood. To improve our knowledge, we integrated transcriptomic data of four studies of human PFC from elderly people (58–80 years old) compared with younger people (20–40 years old) using a meta-analytic approximation combined with molecular signature analysis. We identified 1817 differentially expressed genes, 561 up-regulated and 1256 down-regulated. Pathway analysis revealed down-regulation of synaptic genes with conservation of gene expression of other neuronal regions. Additionally, we identified up-regulation of markers of astrogliosis with transcriptomic signature compatible with A1 neurotoxic astrocytes and A2 neuroprotective astrocytes. Response to interferon is related to A1 astrocytes and the A2 phenotype is mediated in aging by activation of sonic hedgehog (SHH) pathway and up-regulation of metallothioneins I and genes of the family ERM (ezrin, radixin, and moesin). The main conclusions of our study are the confirmation of a global dysfunction of the synapses in the aged PFC and the evidence of opposite phenotypes of astrogliosis in the aging brain, which we report for the first time in the present article.

## 1. Introduction

Aging is the physiological and morphological decline of individuals with the passing of time, which increases their susceptibility to diseases such as cancer, diabetes, neurodegenerative and cardiovascular disorders, and ultimately increases their vulnerability to death. It has become a public health problem since life expectancy has increased, with a consequent world population aging [1].

The brain undergoes functional alterations during aging and the age-related changes do not show a unique pattern across different individuals [2]. Additionally, while some people exhibit characteristics of a healthy aging process, others manifest diminishing motor, sensory and cognitive abilities, with an increased risk of suffering neurodegenerative and neuropsychiatric diseases. The prefrontal cortex (PFC) seems to be morphologically and functionally more vulnerable to the effects of aging compared with others areas [3]. Molecular and cellular responses to aging have been described; for example, neurons show deregulation of transmission, formation, and elimination of synapses. In astrocytes there has been reported an increase of the activation with aging. Recently, genes of reactive astrocytes have been identified to be up-regulated by neuroinflammation in brain mouse [4]. However, the complete molecular mechanisms related to normal human brain aging are not completely understood.

Transcriptomic studies have been successful in identifying some of the specific processes described before [5,6,7,8]. However, they have limitations, such as the inter-individual variability of the aging process, the complexity of getting samples from human brains and the intrinsic technical variation of the transcriptomic methodologies. We hypothesize that combining independent studies by meta-analysis can help us identify the central and common processes associated with PFC aging, avoiding the non-general processes specific of a particular dataset.

We combined by meta-analysis the PFC gene expression profile from two different age groups—58–80 years and 20–40 years—in four independent studies. The selection of these ranges of age was based on the description that, until the forties, gene expression maintains a homogeneous pattern with a low rate of change, and, after this period, the changes begin to rise through several decades to become homogeneous again around the sixties. Bioinformatic analysis of the result of the meta-analysis suggests that in older individuals the neuronal activity declines without necessarily presenting cell death or massive neuron dysfunction. Clusters of genes with presynaptic and postsynaptic functions are down-regulated and over-represented, especially for glutamate, and gamma-aminobutyric acid (GABA) neurons. Additionally, the signature analysis identifies the presence of reactive astrocytes in aged PFC. This astrogliosis is characterized by the presence of up-regulation of genes specific for two different types of reactive astrocytes: A1 and A2. Neurotoxic A1-like astrocytes are recently described in the brain of old rats [4]. However, this is the first time, to our knowledge, that molecular signature of neuroprotective A2 astrocytes are identified in aging of the human PFC.

## 2. Materials and Methods

### 2.1. Data Selection

**Figure 1:**
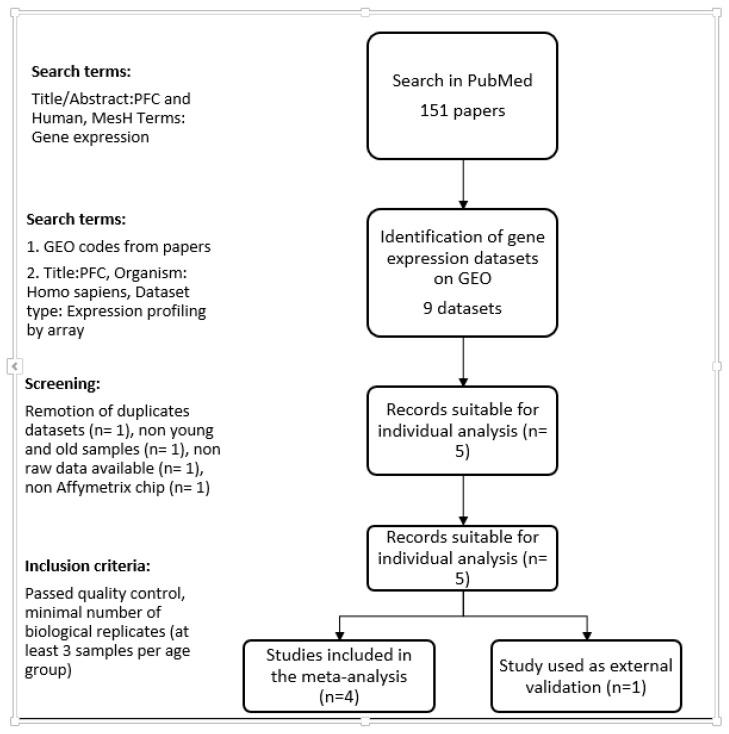
Workflow of the data selection. Search in PubMed and GEO database identified nine datasets involving transcriptomic analysis of prefrontal cortex (PFC) in humans. After removal of duplicates and quality control, five datasets were selected in this research.

We performed an advanced search in the National Center for Biotechnology Information (NCBI) GEO database (http://www.ncbi.nlm.nih.gov/geo/) to identify studies analyzing global gene expression in the human prefrontal cortex. The advanced search tool was used with the keyword PFC (prefrontal cortex) and studies were limited to *Homo sapiens* as the organism, and expression profiling by array as the dataset type. We included studies that met the following conditions: (1) performed using any version of Affymetrix chips; (2) analyzed at least three samples in each age group (elderly: 58–80 years old; young: 20–40 years old); (3) the raw data were available; and (4) passed quality control. We excluded one RNA-Seq study because we wanted to maintain a platform-controlled heterogeneity. The schematic overview of search strategy and selected entries is presented in Figure 1 and the characteristics of the included studies are shown in Table 1.

### 2.2. Quality Control, Batch Effect Adjustment and Data Preprocessing

All datasets underwent quality control (QC) using the QC module from ArrayAnalysis.org [9] to evaluate each microarray. Several parameters were used to detect low-quality samples, including virtual reconstruction of the image, signal comparability and array correlation. Low-quality microarrays were eliminated for the subsequent analysis.

Data preprocessing was performed using limma R/Bioconductor software package [10]. The probesets were summarized, and the data were normalized and then log2 transformed using the RMA algorithm. Since Affymetrix chips have several probes for the same gene, the most informative probe (that one showing the highest variability across the experimental groups) was kept and the others were discarded.

To improve the statistical power and comparability of samples from the same dataset, a batch effect correction was performed using empirical Bayes methods implemented with ComBat [11].

### 2.3. Data Integration by Meta-Analysis

Datasets selected for integration had a similar experimental design, sample size, and chemistry. These datasets were then merged using a modified Fisher’s combined *p*-value meta-analysis implemented through MetaDE R package [12], as was described by Rhodes et al. [13]. For each gene in every dataset, a *p*-value was determined by a *t*-test, after a *p* value modified (*P*-mod) was calculated by multiplying the −log10 (*p*-value) times log1.5 (absolute fold change). Xiao et al. [14] described in detail the *p*-value modification using this methodology. This modification allows the *p*-value to be enriched with the FC magnitude and provides better control of false positives. The *P*-mods of each gene in all datasets were combined using the Rhodes methodology.

### 2.4. Biological Interpretation

DAVID (https://david.ncifcrf.gov/) was used to identify the functions of the selected differentially expressed genes (DEGs). The Kyoto Encyclopedia of Genes and Genomes (KEGG) and gene ontology biological function pathways databases were chosen for the over-representation analysis. Pathways with *p*-values lower than 0.05 were selected as enriched.

### 2.5. Signature Analysis

Over-representation and under-representation analyses were performed using the hypergeometric test as is implemented in the over-representation enrichment analysis described in WebGestalt [15]. Molecular signatures of specific cells, region of cells and molecular phenotypes were mined from the public literature. Signatures were interrogated against lists of DEGs to identify if there are more (over-representation) or fewer (under-representation) genes from the signature in the DEGs than expected by chance.

## 3. Results

### 3.1. Data Selection

After the PubMed and GEO omnibus database search, five studies met the inclusion criteria (Figure 1): GSE53987 [16], GSE11512 [17], GSE17612 [18], GSE17757 [19] and GSE71620 [5]. All selected studies were performed using the Affymetrix platform, in humans, with at least three biological replicates for each experimental group (elderly and young) and they were from different regions of the PFC (Table 1). The first four studies were used to perform the meta-analysis and the last one was used for external validation of the meta-analysis. GSE71620 was selected as validation study because it had many biological replicates. We considered that results obtained by the integrative analysis of several small and independent studies that are concordant with the one single big study imply that the conclusions of the integrative analysis are robust.

### 3.2. Quality Control, Batch Effect Adjustment and Data Preprocessing

**Figure 2:**
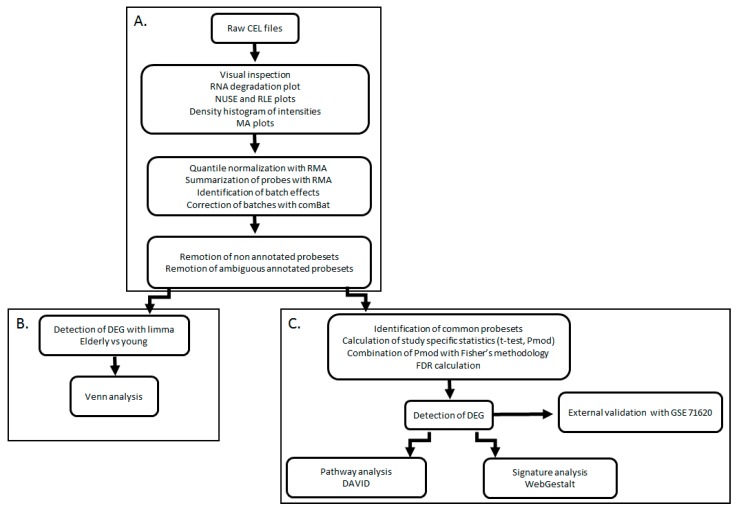
Study workflow. Study was performed in three connected modules. (**A**) Quality control and preprocessing of individual studies. (**B**) Each dataset was analyzed individually using limma and lists of differentially expressed genes (DEGs) were compared by Venn analysis. (**C**) Datasets were combined by meta-analysis. The DEGs were analyzed by pathway over-representation with DAVID and signature analysis was performed using the WebGestalt algorithms. Results of the meta-analysis were validated by comparison with an external dataset (GSE71620).

All arrays involved in the analysis were evaluated for the quality of several parameters (Figure 2). The data quality was determined through RNA degradation ratios, relative log expression and normalized unscaled standard errors using the Arrayanalysis.org platform [9]. Low-quality arrays were removed and the complete datasets were analyzed again to reassess the quality of the remaining samples.

Following this process, a principal component analysis (PCA) plot was calculated for each dataset to detect outliers and identify the unbiased distribution of the samples. Additionally, the scanning date was identified to detect batch effects. A batch correction was performed using ComBat. After batch correction, PCA plots were recalculated to check the modification in the distribution of the samples. All datasets had batch effects and all datasets were batch corrected. Two samples were removed from GSE71620 because there were outliers. Table 1 summarize the studies and samples after the mentioned processes.

### 3.3. Data Integration: Meta-Analysis of Gene Expression in Elderly vs. Young PFC

**Figure 3:**
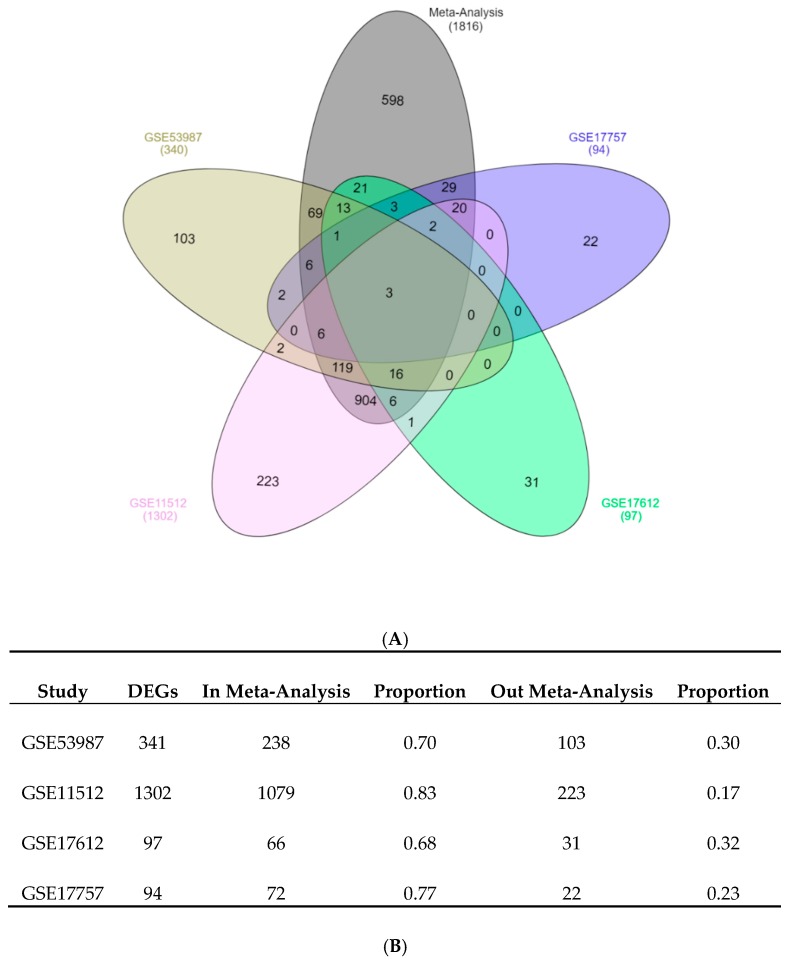
(**A**) Venn diagram of DEGs identified from the individual analysis (GSE11512, GSE17612, GSE53987, and GSE17757) and from the meta-analysis. The meta-analysis identified 1218 genes that were identified by the individual analysis (intersection of the black ellipse with others four ellipses) and 598 additional genes that were not identified in any individual analysis. (**B**) Number and proportion of DEGs from individual analysis and detected by the meta-analysis. “In Meta-analysis” means the DEGs from individual analysis that were preserved in the meta-analysis, while “Out Meta-analysis” means the DEGs from individual analysis that were not present in the meta-analysis.

The meta-analysis methodology used in the integration of the four studies was designed to increase the statistical power of the individual datasets and provided a strong list of DEGs consistently de-regulated across all the comparisons [20]. To determine how the meta-analysis could identify genes that were not recognized by the individual datasets, and to spot the number of genes that were not consistently differentially expressed across the individual analyses, a Venn diagram with the DEGs from each individual analysis and from the meta-analysis was calculated using InteractiVenn [21]. Figure 3 shows the DEGs in each individual analysis compared with the DEGs after the meta-analysis. The meta-analysis identified most of the genes in each individual analysis and was able to detect 598 additional genes. Given that the meta-analysis combined the magnitude of the change in expression, the direction of the change and the level of statistical significance, the detected DEGs had the same direction of change across all datasets in the analysis. A complete list of DEGs is presented in Table S1. When the DEGs were divided into down-regulated and up-regulated genes (Table 2), down-regulated genes outnumbered the up-regulated ones in a ratio of 2.2:1.

Up-regulated genes are genes with an increase in the expression in elderly samples compared with young samples and down-regulated genes are genes with lower expression in elderly samples compared with young samples. Total genes represent the number of genes in each analysis. Common genes are the genes identified simultaneously in both analyses—meta-analysis and GSE71620. The proportion of common genes is the proportion of genes that are common to the meta-analysis and the validation dataset (GSE71620). Those genes had the same direction of change.

The external validation dataset, GSE71620, was analyzed individually with limma: 48 samples from people 60–80 years old were compared with 39 samples from people 20–40 years old to detect the DEGs (Table S2). Out of a total of 18,989 genes, 5120 (27%) had an FDR lower than 0.05. Similar to the meta-analysis findings, there were more down-regulated genes than up-regulated genes (1.5:1). To evidence the reproducibility of the results of the meta-analysis, both lists of DEGs were compared (Table 2). More than 60% of the DEGs were shared between the meta-analysis and GSE71620, and the majority had the same direction of change with aging. Only 19 of 1141 DEGs (2%) had an opposite direction of change. That constitutes a very good overlap between both analyses, as it is usual to find a very small proportion of common DEGs (lower than 10%) when different datasets are analyzed in an independent way [22].

### 3.4. Functional Analysis of Aged PFC

To identify the biological functions of the selected DEGs in the meta-analysis, we performed a pathway analysis in DAVID using KEGG and Gene Ontology Biological process (GOBP) databases. Pathways in KEGG and GOBP have several genes in common. Following this, we used the DAVID cluster tool to identify a set of non-overlapping sets of pathways over-represented in the list of DEGs. To have a better comprehension of the involvement of the pathways in PFC aging, we performed independent analyses with both up-regulated and down-regulated genes. Table 3 shows the pathways over-represented in the down-regulated genes and Table 4 shows the pathways over-represented in the up-regulated genes. Tables S3 and S4 present the list of all genes identified in each pathway.

Down-regulated pathways were located in four clusters. Cluster 1 had 34 genes which were annotated in synapse pathways. Glutamatergic synapses had a higher and more significant proportion of down-regulated genes, followed by dopaminergic and GABAergic synapses. Cholinergic and serotonergic synapses had a lower level of enrichment and higher *p* values. Cluster 2 had 19 genes annotated in the potassium ion transmembrane transport pathway and Cluster 3 was enriched in genes related to inter-cell communication.

Up-regulated pathways were aggregated in four clusters (Table 4). Cluster 1 had 10 genes annotated in mineral absorption, cellular response to cadmium ion and cellular response to zinc ion. Those pathways were over-represented mainly because there were seven metallothionein genes which were up-regulated and annotated in those pathways. Cluster 2 was composed of positive regulation of cellular protein catabolic process and regulation of organelle assembly, among others. It was enriched in genes of the EZR family (ezrin, radixin, and moesin). Cluster 3 contained smoothened signaling pathways, related to the sonic hedgehog (SHH) pathway. Finally, Cluster 4 was enriched in response to the interferon pathway.

### 3.5. Identification of Cell Types Responsible for Aging Changes

PFC is a complex tissue with a combination of several types of cells, and it has been described by previous reports that different PFC cells have different responses in aging [6]. Thus, we wondered which types of cells underwent greater alteration during PFC aging. We hypothesized that cells with major modifications in aging would have an over-representation of cell type specific genes in the list of DEGs. We used the list of specific markers for neurons, oligodendrocytes and astrocytes identified by Cahoy et al. [23] (Table S5) to perform an enrichment signature analysis. The results of the analysis are shown in Table 5. On the list of down-regulated genes from the meta-analysis, there are 1.91 times more down-regulated genes from neurons than expected and 2.04 times fewer down-regulated genes from astrocytes. On the list of up-regulated genes, there are 6.67 times fewer up-regulated genes from neurons than expected and 1.77 times more up-regulated genes from astrocytes. With these results, it is possible to conclude that, in elderly PFC, there is a down-regulation of specific neuron genes and an up-regulation of astrocyte genes. The number of up-regulated and down-regulated DEGs specific to oligodendrocytes were those expected by chance—non-significant *p*-value—meaning that there were no important differences in the function of those cells between elderly and young samples.

### 3.6. Identification of Specific Neuronal Regions with Enrichment of Down-Regulated Genes in Aged PFC

Next, we explored which neuronal zones were more represented in the list of down-regulated genes (Table 6A). To do so, we used well established markers of different zones of the neuron (Table S6). As expected, according to the pathway analysis (Table 3), there were more down-regulated genes from postsynaptic (Enrichment factor, EF = 3.07) and presynaptic (EF = 2.13) regions than expected by chance. Interestingly, markers from other neuronal regions such as the nucleus, cytoplasm, dendritic cytoplasm or axonal cytoplasm were not over-represented. Taken together, these results suggest that, in aging, there is a specific down-regulation of synapses with less alteration in the other neuronal regions.

We discriminate the location of the DEGs annotated in synapses to have a better delineation of the synapse de-regulation (Table 6B). We found that GABAergic synapse and glutamatergic synapse, the two main type of synapses in PFC had alteration in the expression of genes in presynaptic and postsynaptic regions. Interestingly, the other synapses have specific down-regulation of gene expression only in postsynaptic markers (Table S7).

### 3.7. Identification of Pathway Enrichment in Aged PFC Astrocytes

Astrocyte cells had the most over-representation of specific markers in the analysis of up-regulated genes, suggesting that, in aging, there is increased activation of astrocytes. Several studies describe different ways to induce reactive astrogliosis: ischemic stroke (MCAO: middle cerebral artery occlusion) induces activation of astrocytes with a neuroprotective phenotype (A2 astrocytes), while inflammation (LPS: endotoxin LPS from *Escherichia coli* O55:B55) activates astrocytes with neurotoxic properties (A1 astrocytes) [24,25]. Additionally, methamphetamine induces premature senescence [26] and astrocyte activation [27]. We mined the transcriptional signatures of the kinds of activated astrocytes previously described and compared them with our list of DEGs. Table 7 summarizes the results of the over-representation analysis. The A1A2 signature comprises the up-regulated genes in activated astrocytes in general; up-regulated genes in the meta-analysis had 10 times more of those genes than expected. The down-regulated genes did not have any of those genes. These results show that, in aging, not only is there an enrichment of astrocyte markers but those astrocytes are also activated. Signatures for protective (MCAO astrocytes) and detrimental (LPS astrocytes) astrocytes were highly enriched too, with around five times more genes than expected by chance. The methamphetamine signature was not over-represented in either down-regulated or up-regulated genes. The gene signatures used in this analysis can found in Table S6.

Finally, the pathway analysis (Table 4) found that SHH was statistically significantly up-regulated. The SHH is a complex pathway and a recent report indicates that it is important in the interaction among neurons and astrocytes [28]. We analyzed the SHH pathway in aging PFC more deeply. The SHH pathway is modulated by three transcription factors: GLI1/2/3, thus we identified the transcriptional targets of GLI transcription factors using the TF2DNA database [29] (Table S8). To determine if the activation of the SHH pathway was limited to astrocytes, we performed the over-representation analysis with all the transcriptional targets of GLI (GLI total) and using the specific astrocyte genes (GLI astrocyte). GLI1/2/3 target genes were not over-represented in the list of DEGs, but the specific astrocyte GLI1/2/3 targets were over-represented in the list of up-regulated genes with an EF of 3.18, 2.51 and 3.45, respectively (Table 8). Additionally, those lists of genes were under-represented in the list of down-regulated genes.

## 4. Discussion

### 4.1. General Transcriptomic Landscape of Aging in PFC

The combination of elderly PFC vs. young PFC samples from several independent studies by meta-analysis identified a list of DEGs that had high overlapping with the validation dataset constituted by many biological replicates. The approach used in our study could detect genes with a consistent and coherent deregulation across several independent studies. The proportion of down-regulated genes vs. up-regulated genes was 2.2:1. In previous analyses of the aging transcriptomic profile on different tissues and organisms, the number and proportion of down-regulated and up-regulated genes were variable. Meta-analysis of the aged liver in mice found a 1:3 ratio of down-regulated to up-regulated genes [30]. A similar result was obtained in other analyses using several aging organs (kidney, lung, brain cortex, and liver) from humans, mice, and rats [7]. In human lymphoblastoid cells, the proportion was 1:1 [31]. Two studies using whole blood cells found a proportion close to 1.4:1 [32,33]. In a meta-analysis of human muscles, the proportion was 1.2:1 [34]. This variation in the proportion of the direction of de-regulated genes could be explained as follows: since post-mitotic cells (such as muscle cells and neurons) accumulate DNA damage over their lifespan, it is more likely that mutations in transcriptionally active genes will induce a down-regulation, while, mitotically, active cells with an accumulation of mutations are negatively selected and removed from the tissues. Our findings support this hypothesis because the down-regulated genes had an enrichment in specific markers for neurons (post-mitotic cells) and the up-regulated genes had an enrichment in specific markers for astrocytes (cells with proliferative ability in the nervous tissue).

To identify which types of PFC cells were altered in the de-regulation of the transcriptome of elderly samples compared with young samples, we performed on our list of DEGs over-representation and under-representation analyses with specific markers for neurons, oligodendrocytes, and astrocytes [23]. As in previous studies on the cerebral cortex [6], we found an over-representation of neuronal markers in the down-regulated genes and an under-representation of those markers in the up-regulated genes. Astrocyte markers had the opposite over-representation results, with an enrichment of up-regulated genes and fewer down-regulated genes on the list of DEGs. Oligodendrocyte markers had the number of DEGs that would be expected by chance. Taken together, those results indicate that, in aging PFC, there is a down-regulation of neuronal genes without compensatory up-regulation of other neural genes, as well as an increased expression of astrocyte genes. Neuropathological studies show contradictory evidence regarding the change in the number of neurons and neuroglial cells in different regions of the brain with aging. Some results have pointed to a loss of neurons in the rat’s prefrontal cortex [35], basal forebrain [36], thalamus [37], cortex, hypothalamus, cerebellum and olfactory bulb [38]. However, other studies found no modification in the number of neurons in aging. For instance, in the human substantia nigra, there was no correlation between the number of neurons and age [39], and, in the Rhesus macaque, the number of white matter neurons did not show a correlation with age [40]. Therefore, the down-regulation of neuron-specific markers could be explained as a result of a decreased number of neurons or a down-regulation in the expression of genes related to a specific aging phenotype. Likewise, the over-representation of astrocyte genes in the up-regulated genes is due to an increase in the number of cells or their activation.

Our results, as discussed below, support that in aging, there is a down-regulation of gene expression in specific neuronal zones, especially synapses, and opposite patterns of astrocyte activation.

### 4.2. Neuron Transcriptome in Aged PFC: Down-Regulation of Synapses

Specific neuron markers were over-represented in the list of down-regulated genes. The analysis of markers for specific neuron zones evidenced that in aging the more pronounced alteration involved the synapses. Genes that codified proteins from the nucleus and cytoplasm of the neuron body and neuron prolongations were not over-represented. This coincides with a previous analysis using different transcriptomic data [8] and quantitative PCR [41] where the authors reported an altered synaptic gene expression associated with chronological aging. The unbiased analysis of the down-regulated genes showed that all types of synapses were down-regulated, and it is compatible with a general dysfunction of the synaptic connectivity, modulation, and activity. When a careful analysis of the down-regulated genes was performed, a similar down-regulation of presynaptic and postsynaptic genes for GABAergic and glutamatergic synapses was found, indicating a similar involvement of excitatory and inhibitory synapses. Interestingly, for the other types of synapses (serotonergic, cholinergic and dopaminergic), the main down-regulation was almost exclusively restricted to the postsynaptic neuron. In other words, not all synaptic functions were affected equally. For example, genes related to the synthesis and binding of vesicles were down-regulated (SYP, SYT1, SYN2, and STX1A), while genes related with docking and transport of vesicles to the membrane were up-regulated (VAMP1 and SANP23) (Table S1), indicating that the presynaptic dysfunction could be restricted to specific processes.

Even though our research was based on the analysis of transcriptomic datasets, as an additional validation of our results, we found concordant results in a study using quantitative PCR in human elderly PFC [41]. Mohan et al. reported down-regulation of interneuron and synaptic genes (calbindin, somatostatin, cholecystokinin, and SLC17A7), and up-regulation of VAMP1 [41].

### 4.3. Astrocyte Transcriptome in Aged PFC: The Opposite Activation

Astrocytes, the most abundant glial cells, are important for adequate central nervous system (CNS) function. They are involved in the formation and elimination of neuronal synapses [42,43], and also mediate the uptake and recycling of neurotransmitters [44]. We found that, in aging, there is an up-regulation of specific astrocyte markers. These results coincide with a previous report using a different source of information [6]. Current knowledge suggests that astrocyte number is preserved in aging [45,46]. Therefore, the up-regulation of astrocyte markers could be explained by an increase in the activation state of those cells. There are distinctive phenotypes of activated astrocytes, which depend on the stimuli that induce the activation. The best-characterized phenotypes of activated astrocytes are A1 and A2. Reactive astrocytes induced by LPS (A1 astrocytes) exhibit a phenotype that suggests they are detrimental, whereas reactive astrocytes induced by ischemia (A2 astrocytes) exhibit a cellular phenotype that suggests that they are beneficial or protective [24]. The A1 and A2 phenotypes share common genes that are useful for identifying reactive astrocytes in general (activated A1A2 astrocytes). A study in rat brains found an increase of A1-like reactive astrocytes in the hippocampus and striatum with aging [4] suggesting that, in this animal model, astrocyte activation is mainly toxic and it is associated with the loss of brain function. We found that, in elderly PFC, there is a strong up-regulation of A1A2 signature genes. When we analyzed what kind of activated astrocytes were present in elderly PFC, we found a similar over-representation of A1 and A2 signature genes. Additionally, since there are reports linking methamphetamine abuse with the neurochemical profile of aging [47] and premature cellular senescence [26], we compared the molecular profile of the astrocytes activated by methamphetamine abuse with our signature of elderly PFC. However, this profile was not over-represented, indicating that astrocyte activation by methamphetamine is not associated to normal aging astrocyte activation. These joint results indicate that aged human PFC seems to have patterns of gene-expression compatible with heterogeneous astrocyte activation, mixing of protective and toxic astrocyte phenotypes.

Since we used whole tissue with a mixture of cells in our study, we could not delineate more precisely the proportions and specific pathways activated in each type of activated astrocyte. Single cell transcriptomic analysis of astrocytes in aging samples, along with phenotypic analysis of these cells, must be performed to answer this question.

Nonetheless, with the pathway and signature analysis of up-regulated genes, it is possible to suggest the molecular phenotype of astrogliosis in elderly PFC. The fact that mineral absorption was the main up-regulated pathway in the top cluster of activated pathways was an unexpected result of the transcriptomic analysis of the CNS. However, the pathway was statistically significant, because it contained several metallothionein (MT) genes. In the meta-analysis, seven MT genes were analyzed, all of which were from the MT I family, and were up-regulated in elderly PFC. There is an increasing interest in the role of MT in normal and pathological CNS function. The MT superfamily has four isoforms (I–IV); isoforms I and II are expressed in the brain, mainly in astrocytes, while isoform III is expressed in neurons [48]. Metallothioneins I/II are up-regulated in astrocytes in response to neuronal injury [49], and their expression is induced by several stimuli such as metals, hormones, cytokines, oxidative stress and inflammation [50]. The over-expression of MTs is in general protective, for example, when MTs are overexpressed, the mouse lifespan is increased [51]. Metallothioneins I/II play a neuroprotective role in several forms of brain injury and are able to augment the regenerative capacity of astrocytes [52]. These molecules also induce a form of astrogliosis that is permissive with the neurite outgrowth and associated with decreased chondroitin sulfate proteoglycan (CSPG) accumulation. CSPGs are involved in maintaining the structure and function of adult neurons, and in the regulation of proliferation, migration, and neurite outgrowth of neural stem cells in the brain. Aged rats show a significant increase in aggrecan expression throughout the PFC and in the hippocampus [53]. We found up-regulation of the expression of two CSPG genes (BCAN and CD44), and thus the up-regulation of MT I could be related to an astrocyte effort to degrade the increased deposition of CSPGs as a response to synapse malfunction.

Organelle assembly, the second cluster of up-regulated genes, includes the all three ERM family proteins (ezrin, radixin, and moesin). These proteins play a crucial role in organizing membrane domains and regulating signal transduction pathways such as SHH [54]. In the brain, this family is important in the regulation of plasticity and neuroprotection: ezrin (EZR) is required for the structural plasticity of peripheral astrocyte processes associated with synapses [55], moesin (MSN) regulates dendrite arborization and spine-like protrusion growth [56], and radixin (RDX) stimulates adult neural progenitor cell migration and proliferation [57]. Activation of the three members of the family promotes the migration of subventricular zone-derived neuroblasts in response to traumatic brain damage [58]. In elderly PFC, neuronal synapse dysfunction could be sensed by the astrocytes as local damage, and part of the protective response could be the up-regulation of ERM genes. Activation of ERM proteins is mediated by RhoA in HeLa cells [59] and fibroblasts [60], but is independent of RhoA in kidney-derived cells [61]. RhoA was not up-regulated in our analysis nor in previous studies [24] of elderly PFC, but other Rho proteins as RhoJ and RhoU were up-regulated. If those proteins can interact with ERM proteins, then it is plausible that ERM protein activation is caused by other Rho family proteins in the brain and accessory proteins such as ARHGDIA, which is also up-regulated in aging; however, additional analysis of interaction of those proteins are necessary to probe this hypothesis.

Smoothened (SMO) signaling pathway, the representative pathway in the third cluster of up-regulated genes, is the intracellular effector of the activation of Sonic Hedgehog (SHH) pathway. The SHH plays a key role in the development and patterning of the CNS. In the adult brain, SHH is one of the regulators of astrocyte function and activation. Given the importance of this pathway in the biology of astrocytes, we explored in detail their complete regulation in aged PFC. SHH regulates the activity of the GLI transcription factor family, in which there are three members: GLI1, 2 and 3, each with a different role in SHH responsive gene regulation. GLI1 is a transcriptional activator, GLI2 is mainly a transcriptional activator with slight repressor activity, and GLI3 is a transcriptional repressor of target genes [62]. We looked if the transcriptional targets of each GLI were over-represented on the list of DEGs. When all the targets were interrogated, none of GLI targets were over-represented on the list of up-regulated genes and only GLI1 targets were under-represented on the list of down-regulated genes. These results indicate that there is no general deregulation of the SHH pathway in elderly PFC. However, when we selected the GLI targets that are expressed specifically in astrocytes, there was an over-representation of GLI1, 2 and 3 astrocyte targets on the list of up-regulated genes and under-representation of those targets on the list of down-regulated genes. GLI1 and 2 are transcriptional activators and GLI3 is a repressor, thus there is an activation of GLI1 and 2 and inactivation of GLI3 in astrocytes in aging. As a result, there is an overall activation of the SHH pathway, specifically in astrocytes.

Neurons in elderly PFC have a wide down-regulation of expression of synaptic genes, including the genes related to biosynthesis, transport, and release of neurotransmitters. A study recently described that neurons use SHH to control different properties of the astrocytes [28,63]. SHH stimulation of Bergmann glial cells—a type of cerebellar astrocytes—promotes glutamate detection and recovery and potassium homeostasis by up-regulation of SLC1A3 (GLAST) and KCNJ10 (KIR 4.1) [63]. Those genes are up-regulated in elderly PFC, suggesting that the activation of SHH in PFC astrocytes could be a protective response induced by down-regulation in the expression of neuronal synaptic genes. Furthermore, SHH is also involved in neural progenitor proliferation, neovascularization, and synaptogenesis [64]. SHH reduces astrocyte reactivity and the inflammatory response after a brain injury [64], and astrocytes stimulated by SHH protect neurons from cell death [28]. This is compatible with the finding of over-representation of the protective astrocyte signature on the list of up-regulated genes.

On the other hand, we found an over-representation of the neurotoxic astrocyte signature, suggesting that there are parallel pathways of astrocyte activation inducing diverse astrocyte phenotypes in brain aging. Our analysis identified up-regulation of related inflammatory pathways (Cluster 4 of up-regulated genes). This cluster consisted of the enrichment of genes annotated in response to interferon alpha, beta, and gamma. In the aging brain, it is well characterized that interferon signaling at the choroid plexus negatively affects brain function [65] and that the interferon pathways are induced in LPS-reactive astrogliosis [24]. Inflammation is one of the hallmarks of aging, and the hypothalamus integrates inflammatory responses with systemic control of aging through nuclear factor κB (NF-κB) and microglia-neuron neuroimmune crosstalk [66,67]. Inflammation is so important in aging brains that chronic treatment with an IFN-I activator contributes to the development of neurodegenerative disease in wild-type mice [68]. In the context of astrocytes, neurotoxic phenotype development after exposure to LPS is characterized by the induction of interferon pathways [24]. The activation of IFN pathways is also compatible with the aging model that describes inflammatory astrocyte (A1) activation. Moreover, the direct analysis of astrocytes in normal aging showed that one of the up-regulated pathways in mouse old brain astrocytes was interferon signaling [4].

Circadian entrainment pathway was over-represented in the list of down-regulated pathways. Circadian dysfunction is a common symptom of aging, elderly people have a modification in rhythms of behaviors, temperature regulation, and hormone release. Although the samples analyzed in the meta-analysis did not have the time of death available, the meta-analysis identified 28 down-regulated genes annotated in the KEGG pathway of circadian entrainment. The original analysis of the validation dataset GSE71620, which includes this information, found that circadian patterns of gene expression are modified by aging [5]. These results provide an additional validation of our analysis indicating that integrative analysis can identify biologically relevant pathways in aging. The circadian clock is regulated synergistically by neurons and astrocytes [69]. The suprachiasmatic nucleus (SCN) of the hypothalamus coordinates daily rhythms, neurons are in phase with a higher metabolic activity during the circadian daytime, while astrocytes are anti-phasic. They are active during the circadian night and they inhibit neuronal activation by releasing glutamate in the extracellular space [69]. Recently, it is reported that the deletion of Bmal1—one of the master regulators of the molecular clock—induces activation of astrocytes and inflammatory gene expression [70]. The identification in our study of the alteration in the circadian entrainment pathway in the PFC represents one of the possible triggers of the activation of astrocytes in A1-like phenotype in aging.

These results suggest that the up-regulated pathways we found are mainly due to astrocyte activation and they represent two divergent astrocyte molecular and cellular phenotypes of astrogliosis.

## 5. Conclusions

Meta-analysis of transcriptomic data increases the statistical power of the individual datasets and, in addition, can identify DEGs that are consistently de-regulated across the different experiments. A big advantage of this approach is that the particular characteristics of each dataset are masked and only the common processes for all datasets are revealed. In our analysis, we detected that neurons are some of the most important cells affected by aging in the PFC, and, in accordance with other researchers, we delineated the biggest impairment to be in synapse function, with specific variations depending on the type of synapses. Additionally, using the over-representation and under-representation analysis of curated expression signatures, we identified that there are heterogeneous transcriptomic profiles associated with the activation of astrocytes. We found evidence of at least two different phenotypes of activated astrogliosis: A1 (neurotoxic) and A2 (neuroprotective). Due to our analysis design, we could not identify the chronological order or magnitude of those alterations, but the results are consistent with the normal cognitive decline associated with aging. A plausible hypothesis is that neurons, which are post-mitotic cells, accumulated DNA damage for decades, and then they expressed a phenotype characterized by synapse dysfunction. As a response of that, there is activation of astrocytes in at least two different pathways: A1 and A2 astrocytes.

**Figure 4:**
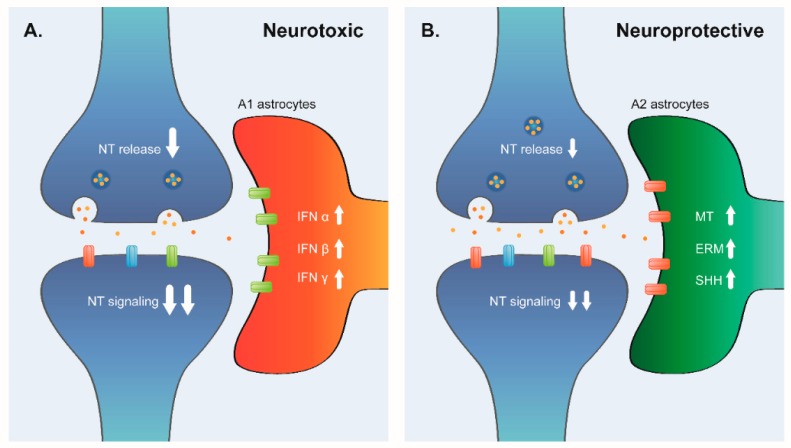
Model of tripartite synapse in elderly PFC. In elderly PFC, there is a down-regulation of expression of presynaptic genes in GABAergic and Glutamatergic synapses and down-regulation of postsynaptic genes in all kind of synapses. They are in two divergent environments (**A**). The presence of A1 astrocytes induces a neurotoxic phenotype; those astrocytes have an activation of inflammatory response represented by interferon pathways. (**B**) There are also A2 astrocytes in elderly PFC. A2 astrocytes have activation of metallothioneins, ERM and SHH pathways. Those pathways are pro-synaptogenic and neuroprotective, thus the alteration in the function of the synapses will be less severe than in (**A**).

We propose a model (Figure 4) where synapses in normal aged PFC are in two states: some synapses are deleteriously related to A1 astrocytes and others are protectively related to A2 astrocytes. A1 astrocytes are the result of activation by aging-related inflammation and A2 astrocytes could be activated as a response to the switch-off of the synapses.

There are several questions remaining: What is the origin of synapse down-regulation? Are the astrocytes phenotypes fixed or can they change with time or stimuli? What is the extent of A1 and A2 activation? How is the local synapse environment under A1 or A2 astrocyte regulatory control? What is the situation of this complex relationship between neurons and reactive astrocytes in neurodegenerative diseases?

Finally, the results of this research are derived from the analysis of bulk tissue and the PFC is composed of a mixture of several types of cells. It is not possible to separate the repercussions of each kind of cells in the transcriptomic deregulation secondary to aging with our experimental setup. The enrichment signature analysis performed in this study gives a global description of the phenomena and can identify the more prominent and consistent biological de-regulated processes. The use of emerging technologies that allow the characterization of the transcriptomic of single cells will be useful to get more in-depth knowledge of the alterations related to aging and to understand in a more detailed way the transcriptomic modifications of astrocytes and neurons in aging.

## Figures and Tables

**Table 1:** Description of studies included in the analysis.

GEO Code	Brain Region	Samples (Elderly/Young)	Platform	Reference
GSE53987	Pre-frontal cortex	4/4	Affymetrix Human Genome U133 Plus 2.0 Array	PMID: 25786133
GSE11512	Dorsolateral prefrontal cortex	4/8	Affymetrix Human Genome U133 Plus 2.0 Array	PMID: 19307592
GSE17612	Brodmann area 10: anterior prefrontal cortex	7/3	Affymetrix Human Genome U133 Plus 2.0 Array	PMID: 19255580
GSE17757	Superior frontal gyrus region of the prefrontal cortex	4/3	Affymetrix Human Gene 1.0 ST Array	PMID: 20647238
GSE71620	Brodmann area 11	48/39	Affymetrix Human Gene 1.1 ST Array	PMID: 26699485

GEO, Gene Expression Omnibus. Young are samples from people 20–40 years old, elderly are samples from people 58–80 years old. GSE71620 was used as an external validation dataset.

**Table 2:** Summary of number and direction of the change of DEGs detected in the analysis.

	Meta-Analysis	GSE71620	Common Genes	Proportion of Common Genes
DEGs	1817	5120	1141	0.63
Up-regulated	561	2076	339	0.60
Down-regulated	1256	3044	783	0.62
Total genes	6895	18989	6895	

**Table 3:** Pathways significantly over represented in down-regulated DEGs in elderly vs. young PFC.

Category	Term	Count	FE	*p*-Value
Cluster 1				
KEGG_PATHWAY	Glutamatergic synapse	34	4.1	1.34 × 10^−12^
KEGG_PATHWAY	Dopaminergic synapse	34	3.7	4.41 × 10^−11^
KEGG_PATHWAY	Circadian entrainment	28	4.1	2.48 × 10^−10^
KEGG_PATHWAY	GABAergic synapse	26	4.3	5.21 × 10^−10^
KEGG_PATHWAY	Cholinergic synapse	24	3.0	2.78 × 10^−06^
KEGG_PATHWAY	Serotonergic synapse	22	2.8	3.25 × 10^−05^
Cluster 2				
GO_BP	Potassium ion transmembrane transport	19	2.4	9.52 × 10^−04^
Cluster 3				
KEGG_PATHWAY	Circadian entrainment	28	4.1	2.48 × 10^−10^
KEGG_PATHWAY	Oxytocin signaling pathway	26	2.3	1.34 × 10^−04^
KEGG_PATHWAY	cGMP-PKG signaling pathway	25	2.1	7.13 × 10^−04^
KEGG_PATHWAY	Long-term depression	13	3.0	9.54 × 10^−04^
KEGG_PATHWAY	Gap junction	16	2.5	0.001
KEGG_PATHWAY	Inflammatory regulation of TRP channels	16	2.3	0.004
Cluster 4				
KEGG_PATHWAY	Nicotine addiction	15	5.2	3.27 × 10^−07^

The pathways were clustered by genes in common. *p*-values were corrected by multiple comparisons with the DAVID methodology. Count is the number of down-regulated DEGs. FE is the fold enrichment, i.e., the additional times there are more DEGs in the pathway than expected by chance.

**Table 4:** Pathways significantly over represented in DEGs up-regulated in elderly vs. young meta-analysis.

Category	Term	Count	FE	*p*-Value
Cluster 1				
KEGG_PATHWAY	Mineral absorption	10	6.1	2.76 × 10^−05^
GO_BP	Cellular response to cadmium ion	6	11.5	1.19 × 10^−04^
GO_BP	Negative regulation of growth	6	10.3	2.13 × 10^−04^
GO_BP	Cellular response to zinc ion	6	10.3	2.13 × 10^−04^
Cluster 2				
GO_BP	Positive regulation of cellular protein catabolic process	4	11.9	0.004
GO_BP	Regulation of organelle assembly	3	24.5	0.005
GO_BP	Positive regulation of protein localization to early endosome	3	19.6	0.009
GO_BP	Establishment of endothelial barrier	4	8.2	0.01
GO_BP	Positive regulation of early endosome to late endosome transport	3	12.3	0.023
Cluster 3				
GO_BP	Negative regulation of smoothened signaling pathway	5	8.6	0.002
GO_BP	Dorsal/ventral pattern formation	6	6.1	0.003
GO_BP	Smoothened signaling pathway	6	2.8	0.059
Cluster 4				
GO_BP	Response to interferon-beta	4	14.5	0.002
GO_BP	Response to interferon-gamma	5	6.8	0.006
GO_BP	Negative regulation of viral genome replication	5	4.1	0.031
GO_BP	Response to interferon-alpha	3	9.8	0.036

The pathways were clustered by genes in common. *p*-values were corrected by multiple comparisons with the DAVID methodology. Count is the number of up-regulated DEGs. FE is the fold enrichment, i.e., the additional times there are more DEGs in the pathway than expected by chance.

**Table 5:** Signature analysis of specific markers of neuron, oligodendrocyte and astrocyte.

	Down-Regulated		Up-Regulated	
ID	EF	*p*-Value	EF	*p*-Value
Neuron	1.91	0.00 × 10^+00^	−6.67	0.00 × 10^+00^
Oligodendrocyte	−1.33	1.00 × 10^+00^	−1.15	9.56 × 10^−01^
Astrocyte	−2.04	0.00 × 10^+00^	1.77	0.00 × 10^+00^

EF means enrichment factor, which is the number of times that there were more genes down-regulated or up-regulated than expected by chance. Positive EF means there is an over-representation of genes of cell type, while negative EF means an under-representation of genes of cell type.

**Table:** (**A**)

	Down-Regulated	
ID	EF	*p*-Value
Neuron Postsynaptic	3.07	2.26 × 10^−02^
Neuron Presynaptic	2.13	4.30 × 10^−02^
Neuron Dendritic Axonal Cytoplasmic	1.73	2.41 × 10^−01^
Neuron Nuclear Cytoplasmic	−1.09	6.73 × 10^−01^
Growth Cone Markers	−2.38	9.32 × 10^−01^

**Table:** (**B**)

	Presynaptic	Postsynaptic	Total
GABAergic synapse	15	24	26
Glutamatergic synapse	19	20	34
Dopaminergic synapse	0	34	34
Serotoninergic synapse	0	21	22
Cholinergic synapse	7	24	24

**Table 7:** Signature analysis of several signatures of activated astrocytes.

	Down-Regulated		Up-Regulated	
ID	EF	*p*-Value	EF	*p*-Value
A1A2	NA	NA	10.13	1.99 × 10^−06^
LPS astrocyte (A1)	−4.76	0.004	4.83	2.82 × 10^−05^
MCAO astrocyte (A2)	−2.63	0.021	5.41	7.96 × 10^−07^
Methamphetamine	1.21	0.288	−1.79	0.113

A1A2 is the signature of astrocyte activation. EF means enrichment factor, which is the number of times that there are more genes down-regulated or up-regulated than expected by chance. Positive EF means there is an over-representation of genes of cell type, negative EF means an under-representation of genes of cell type.

**Table 8:** Signature analysis of targets of GLI transcription factors in DEGs.

	Down-Regulated		Up-Regulated	
ID	EF	*p*-Value	EF	*p*-Value
GLI1 astrocyte	−1.69	0.013	3.18	2.59 × 10^−07^
GLI1 total	1.12	0.034	1.02	0.471
GLI2 astrocyte	−2.17	0.001	2.51	0.001
GLI2 total	1.04	0.306	1.2	0.953
GLI3 astrocyte	−2.63	0.021	3.45	0.002
GLI3 total	1	0.528	−1.14	0.258

GLI1 total means that all the transcriptional targets of GLI1 were interrogated against the list of DEGs. Similarly, GLI2 and GLI3 mean the transcriptional targets of the same transcription factor. GLI1 astrocyte, GLI2 astrocyte and GLI3 astrocyte mean that only the transcriptional targets present in the list of specific markers of astrocyte were used.

## Supplementary Materials

The following are available online at http://www.mdpi.com/2076-3425/8/12/227/s1, Table S1. Results of the meta-analysis. It shows the result of the *P*-mod value for down-regulated and up-regulated genes for all genes analyzed. Table S2. Results of the external validation. It shows the adjusted *p*-value after the comparison of elderly samples with young samples from GSE71620. Column meta-analysis shows the direction of change in meta-analysis: down-regulated, Down-; up-regulated, Up-; without change, NoChange; if it was not present in the meta-analysis, No present. Table S3. Pathway analysis of down-regulated genes. Pathways over-represented in the list of down-regulated genes as detected by DAVID. Clusters represent pathways with overlapping genes. Table S4. Pathway analysis of up-regulated genes. Pathways over-represented in the list of up-regulated genes as detected by DAVID. Clusters represent pathways with overlapping genes. Table S5. List of molecular signatures for neurons, astrocytes and oligodendrocytes. Specific markers of PFC cells: neurons, astrocytes and oligodendrocytes. Meta-analysis column informs if the gene was: down-regulated, Down; up-regulated, Up; without change, No Change; if it was not present in the meta-analysis, No present. Table S6. Neuron zone signatures and astrocyte signatures. List of markers of specific zones of neurons, and different astrocyte molecular phenotypes. Table S7. Localization of synaptic DEGs. List of DEGs annotated in synapses and description of the localization of the product of the gene in the presynaptic or postsynaptic zones according KEGG. Table S8. Transcriptional targets of GLI transcription factors. List of genes annotated as transcriptional targets of GLI1/2/3 in TF2DNA.
